# Diminution of Phagocytosed Micro/Nanoparticles by Tethering with Immunoregulatory CD200 Protein

**DOI:** 10.1038/s41598-020-65559-z

**Published:** 2020-05-25

**Authors:** Jun Zhang, Ching-An Peng

**Affiliations:** Department of Biological Engineering, University of Idaho, Moscow, ID 83844 USA

**Keywords:** Bioinspired materials, Isolation, separation and purification, Drug delivery

## Abstract

CD200 is known as an anti-inflammatory transmembrane glycoprotein in the immunoglobulin superfamily. CD200 interacts with its receptor CD200R which is highly expressed on myeloid cells such as macrophages and neutrophils. CD200-CD200R interaction has known to reduce macrophage activation and chronic inflammation. To harness the immunomodulatory property of CD200 for surface modification, CD200-streptavidin fusion protein was expressed from bacteria transformed with pET20b plasmid encoded with CD200 extracellular domain and core streptavidin. The purified CD200-SA protein was bound to biotin-coated fluorescent polystyrene particles of various sizes ranging from 0.15 to 2 µm. THP-1 macrophages were cultivated with CD200-modified micro/nanoparticles in comparison with controls. Our results showed that both nano- and micro-sized particles decorated with CD200 decreased phagocytosis activities of THP-1 macrophages. Such diminution of phagocytosis was examined to be associated with downregulation of Toll-like receptor 4 (TLR4) expression on the surface of macrophages. Moreover, THP-1 macrophages treated with CD200-coated particles decreased the secretion of tumor necrosis factor-α (TNF-α).

## Introduction

Macrophage is a phagocyte of the immune system that engulfs foreign microbes and cellular debris^1^. Macrophage clearance is a major challenge for therapeutic application of particles because micro/nanoparticles are quickly cleared by macrophages^2–4^. The surfaces of particles have been modified by various materials to reduce phagocytic clearance by macrophages. Traditionally, modification with polyethylene glycol (PEG) hydrophilic chains reduces the macrophage uptake by making a “stealth” surface on the particles and thus prolonging their circulation time^5,6^. Although surface modification with PEG achieves longer circulation time in the blood, PEGylation may have some drawbacks such as attenuated delivery efficiency, immunological responses and non-biodegradability^7–9^.

CD200 is an anti-inflammatory transmembrane glycoprotein in the immunoglobulin superfamily. CD200 is expressed in a myriad of cells such as myeloid cells (macrophages, dendritic cells), lymphoid cells (T, B cells), neurons, cardiomyocytes, keratinocytes, and endothelial cells^10–12^. CD200R known as CD200 cognate receptor is expressed on the surface myeloid cells such as macrophages, neutrophils, and microglia^13,14^. Hoek *et al*. found that CD200-CD200R interaction elicits an inhibitory signal that downregulates macrophage activity^13^. In cancer stem cell (CSC) immunology, the expression of CD200 on CSCs allows the CSCs to evade or suppress the immune system^15^. Several other studies also supported the inhibitory role of CD200-CD200R signal in the activation of macrophages and dendritic cells^16–19^. Most functional findings of CD200-CD200R interaction have focused on dampening pro-inflammatory cytokine secretion of myeloid cells. More recently, the involvement of CD200-CD200R interaction with decreased myeloid cell phagocytosis has been reported. For example, *in vivo* CD200-Fc administration facilitated white matter recovery by suppressing phagocytosis of susceptible oligodendrocyte precursors^20^. The absence of CD200-CD200R interaction led to increased engulfment of FITC-labelled amyloid-β or fluorescently labelled 1-μm latex beads in microglial cells isolated from CD200^−/−^ mice. Moreover, increased phagocytosis was observed in microglial cells isolated from CD200-deficient mice^21^. In contrast, a study indicated that CD200 coating of 7 μm fluorescein-containing poly(lactic-co-glycolic acid) (PLGA) microparticles enhanced their phagocytosis by both mouse macrophages and human monocytes^22^. Since the PLGA microparticles were synthesized by that lab (potential with high polydispersity) and the heterogenous distribution of CD200 on the microparticles might lead to low number of CD200 sensing CD200R on the surface of macrophages, the claimed increment in phagocytosis from 8 to 15% could be a concern. Moreover, the very low phagocytic rate was probably because the 7-μm PLGA particles were not easily engulfed by macrophages. Judging from the inconsistence of CD200-CD200R engagement on the ingestion of CD200-coated particles by phagocytes, this topic warrants further investigation with particles having less polydispersity for uniform distribution of CD200 and small sizes for easier taken up by macrophages.

In the present study, the surfaces of polystyrene particles with sizes ranging from nano- to micro-meters were coated with CD200 to evaluate the effect of CD200 on macrophage phagocytosis. We hypothesized that surface modification of micro/nanoparticles would decrease macrophage phagocytosis as well as the secretion of pro-inflammatory cytokines through CD200-CD200R interaction. The CD200 protein used here is tagged with streptavidin (SA) by recombinant DNA approach. The expressed and purified CD200-SA fusion protein was bound to biotinylated fluorescent polystyrene particles via high affinity of SA with biotin. THP-1 macrophages were treated with CD200-coated and control particles. The antiphagocytic efficacy of CD200 was evaluated. Our results showed that both nano- and micro-sized particles decorated with CD200 decreased phagocytosis activities of THP-1 macrophages. Moreover, THP-1 macrophages treated with CD200-coated 0.56 µm particles showed 26.9% and 26.1% decrease in interleukin-6 (IL-6) and tumor necrosis factor-α (TNF-α) secretion, respectively. Surface modification with CD200 can be potentially used as an approach to avoid phagocytic clearance by macrophages in order to prolong the circulation time of drug delivery carriers and thus promoting delivery of drug to target sites.

## Materials and Methods

### Materials

Plasmid encoding human CD200 DNA was acquired from DNASU Plasmid Repository of Arizona State University (plasmid ID No: HsCD00620970; Tempe, Arizona, USA). Phusion DNA polymerase and Quick-Load Purple 2-Log DNA Ladder were purchased from New England Biolabs (Ipswich, MA, USA). FastDigest restriction enzymes (XhoI, EcoRI, BamHI and EcoRV), T4 DNA ligase, subcloning efficiency DH5α competent cells, glycerol, B-PER bacterial protein extraction reagent, Halt protease inhibitor, lysozyme, DNase I, HisPur Ni-NTA Resin, disposable polystyrene columns, Coomassie brilliant blue R 250, Pierce ECL Western blotting substrate, Tween-20, BCA protein assay kit and RPMI 1640 Medium, human IL-6 ELISA kit and human TNF-α ELISA kit were purchased from Thermo Fisher Scientific (Waltham, MA, USA). Plasmid Maxi Kit was purchased from QIAGEN (Valencia, CA, USA). Ampicillin, Isopropyl β-D-1-thiogalactopyranoside (IPTG), penicillin-streptomycin, phorbol 12-myristate 13-acetate (PMA), fluorescein isothiocyanate (FITC), biotin-FITC, 1-ethyl-3-(3-dimethylaminopropyl)carbodiimide (EDC), zymosan from *Saccharomyces cerevisiae* and sodium bicarbonate were purchased from Sigma-Aldrich (St. Louis, MO, USA). Rosetta (DE3) competent cells were purchased from Merck Millipore (Darmstadt, Germany). Acetic acid was purchased from J.T. Baker (Philipsburg, NJ, USA). Imidazole, sodium chloride (NaCl) was purchased from ACROS (Fair Lawn, NJ, USA). Laemmli sample buffer was purchased from Bio-Rad (Hercules, CA, USA). Methanol was purchased from Avantor Performance Materials (Center Valley, PA, USA). Polyvinylidene difluoride (PVDF) membrane was purchased from Pall Life Science (Port Washington, NY, USA). Human/mouse CD200 antibody and mouse IgG HRP-conjugated antibody were purchased from R&D Systems (Minneapolis, MN, USA). Rabbit anti-streptavidin antibody, goat anti-rabbit IgG horseradish peroxidase conjugate were purchased from GenScript (Piscataway, NJ, USA). Bovine serum albumin (BSA) was purchased from Rockland Immunochemicals Inc (Limerick, PA, USA). THP-1 cell line (catalog number TIB-202) was purchased from ATCC (Manassas, VA, USA). Fetal bovine serum (FBS) was purchased from Atlanta Biologicals (Lawrencevilla, GA, USA). Green fluorescent (diameter = 0.15 µm) and red fluorescent (diameter = 0.56, 0.84, 2 µm) biotin-coated fluorescent polystyrene particles were purchased from Spherotech (Lake Forest, IL, USA). 2-mercaptoethanol was purchased from Gibco (Grand Island, NY, USA). 2-(N-morpholino)ethanesulfonic acid (MES) was purchased from TCI (Portland, OR, USA). Trypan blue was purchased from BTC Beantown Chemical (Hudson, NH, USA). Anti-human CD200 receptor monoclonal antibody with PE label was purchased from BioLegend (San Diego, CA, USA). Mouse IgG isotype control and human toll-like receptor 4 (TLR4) antibody (HTA125) with FITC label was purchased from Santa Cruz Biotechnology (Dallas, Texas, USA).

### Construction of CD200-SA encoding plasmid

**Figure 1:**
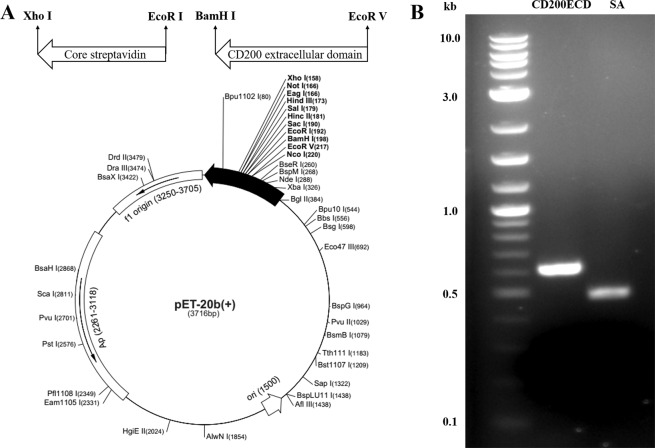
(**a**) The circular map showing multiple cloning sites for CD200-SA fusion gene. CoreSA was inserted between XhoI and EcoRI; the extracellular domain of human CD200 was inserted between BamHI and EcoRV. (**b**) DNA gel electrophoresis of DNA ladder (lane 1), extracellular domain of human CD200 (~0.6 kb) (lane 2) and coreSA (~0.5 kb) (lane 3).

The CD200-SA encoding recombinant plasmid (pMZ006) was constructed by inserting the gene sequences of CD200 extracellular domain (CD200ECD) and core streptavidin (coreSA) into plasmid vector pET20b (Fig. 1a). The extracellular domain of CD200 was amplified from full-length human CD200 by polymerase chain reaction (PCR) with sense primer 5′-TAAGTCGATATCGGTCATGGCAGCAGTGGT-3′ and antisense primer 5′-TAAGCAGGATCCCACCTGGCAGATCACCTC-3′. CD200 PCR reactions were performed with Phusion DNA polymerase in a thermocycler (Bio-Rad, Hercules, CA, USA) with initial denaturation at 98 °C for 30 s, and then 30 cycles of denaturation at 98 °C for 10 s, annealing at 62 °C for 30 s, and extension at 72 °C for 15 s. Final extension was performed at 72 °C for 5 min. CoreSA DNA was amplified by PCR from pSTE2-215 (yol) plasmid^23^ using sense Primer: 5′-ACTGGAATTCGCTGAAGCAGGTATCACC-3′ and antisense primer: 5′-ATCGCTCGAGGATGGGATAGATCTTCTTCTG-3′. CoreSA PCR reactions were performed with Phusion DNA polymerase in a Bio-Rad thermocycler with initial denaturation at 98 °C for 30 s, and then 30 cycles of denaturation at 98 °C for 10 s, annealing at 60 °C for 30 s, and extension at 72 °C for 15 s. Final extension was performed at 72 °C for 5 min. The PCR products of CD200 and coreSA were analyzed by 1% agarose gel electrophoresis. pET20b plasmid (vector) and coreSA DNA (insert) were cut with restriction enzymes XhoI and EcoRI. The digested pET20b and coreSA were ligased by T4 DNA ligase to get PMZ005 plasmid. The ligation product PMZ005 was amplified by transformation with subcloning efficiency DH5α competent cells and purified by QIAGEN Plasmid Maxi Kit. The PMZ005 plasmid (vector) and CD200 DNA (insert) were cut with restriction enzymes BamHI and EcoRV. The digested PMZ005 and CD200 products were ligased by Blunt/TA Ligase Master Mix to get PMZ006 plasmid. The PMZ006 ligation product was again amplified by transformation with subcloning efficiency DH5α competent cells and purified by QIAGEN Plasmid Maxi Kit.

### Expression and purification of CD200-SA fusion protein

Rosetta (DE3) competent cells were transformed with CD200-SA encoding plasmid PMZ006 and grown on agar plates containing 50 μg mL^−1^ ampicillin. A single colony was picked up and grown in 5 mL of lysogeny broth (LB) medium supplemented with 10% (v/v) glycerol and 50 μg mL^−1^ ampicillin at 225 rpm, at 37 °C in a shaker incubator for overnight. The start culture was then diluted 1:20 with LB containing 10% (v/v) glycerol and 50 μg mL^−1^ ampicillin, and grown in a shaker incubator at 225 rpm, 37 °C. The optical density (OD) at 600 nm was monitored by a Spectramax M2e microplate reader (Molecular Devices, Sunnyvale, CA, USA). When OD 600 nm reached 0.6 to 0.8, IPTG was added to the culture to the final concentration of 0.4 mM to induce the protein expression. The culture was grown in a shaker incubator at 225 rpm at 37 °C for 2 h. The cells were harvested by centrifugation at 4,500 rpm for 5 min at 4 °C. The cell pellet was washed with cold 1× phosphate buffer saline (PBS), and harvested at 4,500 rpm for 5 min at 4 °C.

The cell pellet from 50 mL bacterial culture was resuspended in 5 mL of B-PER bacterial protein extraction reagent supplemented with 1× Halt protease inhibitor, 1 mg mL^−1^ lysozyme, and 2 µL mL^−1^ DNase I. Cell lysis was performed according to manufacturer’s instruction to collect the clear protein lysate. 1 mL of protein lysate was mixed with 1 mL of binding buffer (50 mM Na_2_HPO_4_, 300 mM NaCl, pH 7.4), and then applied to an IMAC column filled with 1 mL of HisPur Ni-NTA resin. The resin was then washed with one resin-bed volume (1 mL) of wash buffer (10 mM imidazole, 50 mM Na_2_HPO_4_, 300 mM NaCl, pH 7.4) 5 times or until the absorbance at 280 nm reached baseline. CD200-SA fusion protein was eluted with 5 resin-bed volumes of elution buffer (250 mM imidazole, 50 mM Na_2_HPO_4_, 500 mM NaCl, pH 7.4). Protein elution was monitored by measuring absorbance at 280 nm. To determine the concentration of purified CD200-SA protein, BCA protein assay kit was used according to the manufacturer’s instructions. Briefly, 2 mg mL^−1^ BSA standard was serially diluted and used as protein standards. BCA reagent A was mixed with BCA reagent B at 50:1 volume ratio, and the mixture was used as working reagent. 10 μL of purified protein was mixed with 200 μL of working reagent and incubated at 37 °C for 30 min. The absorbance at 562 nm was measured by a Spectramax M2e microplate reader.

### SDS-PAGE and Western blot analysis

Both crude proteins from bacterial lysate and purified CD200-SA protein were characterized by SDS-PAGE. The protein lysate was mixed with same volume of Laemmli sample buffer and loaded on a 12% polyacrylamide gel made by Mini-PROTEAN Tetra handcast systems (Bio-Rad). SDS-PAGE was performed at 200 V for 40 min. The gel was stained with 0.006% (w/v) Coomassie brilliant blue R 250 solution with 10% (v/v) acetic acid and 40% (v/v) methanol. The gel was destained in a fast destaining solution containing 10% (v/v) acetic acid and 40% (v/v) methanol for 30 min, and followed by slow destaining in a solution containing 10% (v/v) acetic acid and 10% (v/v) methanol for overnight.

For Western blot analysis, purified CD200-SA protein on the 12% polyacrylamide gel was transferred to a PVDF membrane using a Trans-Blot SD semi-dry transfer cell (Bio-Rad) at 18 V for 1 h. The membrane was washed with 1X Tris buffered saline (TBS) at room temperature for 10 min, and then blocked with blocking buffer (5% (w/v) BSA in TBS with 0.1% Tween-20) at room temperature for 1 h with mild shaking. It should be noted that BSA was chosen to make the blocking buffer because non-fat dry milk contains biotin and it interfered with the detection of coreSA protein. The membrane was then immersed in 1:500 diluted human/mouse CD200 antibody solution in blocking buffer, and incubated on a mini rocker (Hercules, CA, USA) at 4 °C for overnight. The membrane was washed with Tris-buffered saline, 0.1% Tween 20 (TBST) for 10 min, three times at room temperature, and then incubated with 1:1,000 diluted mouse IgG HRP-conjugated antibody for 1 h at room temperature. The membrane was washed with TBST for 10 min, three times. The CD200 protein was characterized by detecting horseradish peroxidase activity using ECL substrate via chemiluminescence imager (PXi, Syngene, MD, USA). Similarly, coreSA was detected by rabbit anti-streptavidin antibody as the primary antibody and goat anti-rabbit IgG horseradish peroxidase conjugate as the secondary antibody.

### Preparation and characterization of CD200-coated fluorescent particles

Biotin-coated fluorescent polystyrene particles of various sizes (0.15, 0.56, 0.84 and 2 µm) were suspended in purified CD200-SA protein solution for biotin-SA binding. The particles were gently shaken at 4 °C for 30 min, and particles were collected by centrifugation at 8,000 × g for 5 min, followed by three times wash with 1× PBS. Surface modification of 0.56 µm particles with CD200 was characterized by dot blot analysis. Briefly, 2 µL of CD200-modified 0.56 µm particles and 2 µL of unmodified particles were added to a PVDF membrane. The membrane was blocked with blocking buffer (5% (w/v) BSA in TBST at room temperature for 1 h with mild shaking. The membrane was then incubated in primary antibody (human/mouse CD200 monoclonal antibody) and secondary antibody (mouse IgG HRP-conjugated antibody) solutions described similarly in the previous section. The membrane was also treated with ECL substrate and imaged by chemiluminescence imager same as described in the previous section.

Both biotin and CD200 modified polystyrene particles (with the sizes of 0.15, 0.56, 0.84 and 2 µm provided by the vendor) were resuspended in RPMI 1640 medium supplemented with 10% FBS. Hydrodynamic diameters and zeta potentials of measured using a Brookhaven ZetaPALS Zeta Potential Analyzer (Brookhaven Instruments, Holtsville, NY, USA). The prepared CD200-coated particles were stored in RPMI 1640 medium supplemented with 10% FBS at 4 °C for 72 h, and hydrodynamic diameters and zeta potentials were measured again after 72 h to examine stability of the prepared CD200-coated particles. To quantify CD200-SA coated on 0.15, 0.56, 0.84 and 2 µm polystyrene particles, 200 µL of 0.15, 0.56, 0.84 and 2 µm biotinylated polystyrene particles (0.1% w/v) was dissolved in 1 mL purified CD200-SA protein and mildly shaken for 30 min. The particles were collected by centrifugation at 8,000 × g for 5 min, followed by washing with 1× PBS for three times. CD200-SA coated particles were then resuspended in a solution containing biotin-FITC and gently shaken for 30 min. After biotin-FITC bound CD200-SA coated particles, the particles were washed and resuspended in 100 µL 1× PBS. The FITC intensity (λ_ex_ = 485 nm, λ_em_ = 525 nm) was measured by a Spectramax M2e microplate reader. The molar concentration of CD200 was quantified using the standard calibration curve of biotin-FITC. The amount of CD200 on particles was expressed as molecules µm^−2^. It should be noted that three different batches of polystyrene particles and purified CD200-SA protein were used for the preparation of CD200-coated polystyrene particles.

Fluorescent zymosan particles (size ~ 3 µm) were also prepared to test the effect of CD200 on macrophage phagocytosis. The preparation of fluorescent zymosan particles was previously described^24^. 10 mg of zymosan suspended in 10 mL of 0.3 M sodium bicarbonate buffer (pH 9.2). The particles were centrifuged and resuspended to 5 mL MES buffer (pH 7.4). The particles were then tip-sonicated 30 sec for three times. 50 µL of 1 mg mL^−1^ FITC was added to the zymosan particles and incubated for overnight. The excess FITC was washed by 1× PBS for three times. 10 mg biotin and 10 mg EDC were dissolved in 10 mL MES buffer (pH 7.4) and stirred for 15 min. The FITC-labeled zymosan particles were added to the biotin-EDC mixture and stirred for 3 h at room temperature. The zymosan-biotin-FITC particles were collected by centrifugation and washed by 1× PBS for three times.

### Phagocytosis study of THP-1 macrophages

THP-1 macrophages were cultured with RPMI 1640 medium supplemented with 10% FBS, 1% penicillin-streptomycin and 0.05 mM 2-mercaptoethanol. The differentiation of THP-1 cells to macrophages was induced by supplementing the culture medium with 1.6 × 10^−7^ M PMA for 2 days. The seeding density was 5 × 10^4^ cells cm^−2^ on 6-well plates. After 2 days, medium was discarded and cells were washed with 1× PBS for three times.

The expression of CD200R on THP-1 macrophage surface was quantified by flow cytometry (FACS) study. THP-1 macrophages were detached from culture flasks by gentle scraping. Cells were collected by centrifugation at 400 × g for 5 min, followed by washing with 1× PBS. PMA-induced THP-1 macrophages were first reacted with mouse IgG isotype control for 30 min, and then incubated in 1× PBS with 3% BSA containing human CD200R antibody with PE label for 30 min. Cells were washed three times by centrifugation at 400 × g for 5 min. The cells were analyzed by BD Accuri C6 flow cytometer (BD Biosciences, San Jose, CA, USA), and data was processed by BD Accuri C6 Plus software. The effect of CD200-coated particles on the expression of TLR4 on macrophage surface was also evaluated by FACS. THP-1 macrophages were allowed to engulf unmodified and CD200-coated 0.56 µm particles (Nile red) for 2 h. The cells were detached from the culture flasks by gentle scraping. Cells were collected by centrifugation at 400 × g for 5 min, followed by washing with 1× PBS. PMA-induced THP-1 macrophages were first reacted with mouse IgG isotype control for 30 min, and then incubated in 1× PBS with 3% BSA containing human TLR4 monoclonal antibody (HTA125) with FITC label for 30 min. Cells were washed three times by centrifugation at 400 × g for 5 min. The cells were analyzed the same as described above.

To assess the effect of CD200 on macrophage phagocytosis, THP-1 macrophages were inoculated on plate wells at the seeding density of 5 × 10^4^ cells cm^−2^. THP-1 macrophages were treated with CD200-coated and unmodified fluorescent polystyrene particles of various sizes for 2, 5, and 10 h. To further confirm the interaction of CD200-CD200R results in the antiphagocytic signal, inhibition assay was performed by blocking CD200-coated polystyrene particles with anti-CD200 antibody. Briefly, 100 µL solution of CD200-coated particles was mixed with 3 µL anti-CD200 antibody for 30 min. The CD200-coated particles blocked with anti-CD200 antibody were centrifuged at 8,000 × g for 5 min, followed by washing with 1× PBS for three times. The particles added to THP-1 macrophages ranged from ~50 to 5 × 10^3^ particles per cell; that is, roughly 50, 250, 500, and 5000 particles per THP-1 macrophage cell for polystyrene particles with size of 2, 0.84, 0.56, and 0.15 µm, respectively. It should be noted that, for a given size of unmodified and CD200-coated polystyrene particles, cells were treated with the same number of particles per cell. At each time point, the particles not taken up by macrophages were gently washed away with 1× PBS for three times, and then quenched by treating cells with 0.02 mg mL^−1^ trypan blue. Furthermore, 0.56 µm particles were specifically modified with SA (expressed from pMZ005-transformed competent cells) to compare with the effect of CD200 (expressed from PMZ006-transformed competent cells) on macrophage phagocytosis. Bright field and fluorescent images were taken with Leica DMi8 microscope equipped with Leica EC3 camera (Leica Microsystems, Wetzlar, Germany). The percentage of macrophage phagocytosis was calculated by taking the average counts of 10 microscopic images, according to the following equation:

1 $$ \% \,of\,phagocytosis=\frac{Number\,of\,cells\,ingesting\,fluorescent\,particles}{Number\,of\,total\,cells}$$

To avoid any potential error made by the human factor, the fluorescence intensities of macrophages engulfing unmodified particles, CD200-coated particles, and CD200-coated particles blocked with anti-CD200 antibody were measured by a Spectramax M2e microplate reader. The excitation and emission wavelengths were λ_ex_= 485 nm, λ_em_=525 nm for yellow and green fluorescence, and λ_ex_ = 552 nm, λ_em_ = 636 nm for Nile red and pink fluorescence.

### The effect of CD200-coated on IL-6 and TNF-α secretion from THP-1 macrophages

THP-1 macrophages were seeded on 6-well plates at 5 × 10^4^ cells cm^−2^. THP-1 macrophages were treated with unmodified and CD200-coated 0.56 µm polystyrene particles (Nile red) for 18 h. The level of IL-6 and TNF-α in culture media was quantified by commercial ELISA kits according to manufacturer’s instructions. Briefly, 100 µL coating buffer containing IL-6 and TNF-α capture antibody was added to each well of the ELISA 96 well plate and incubated overnight at 4 °C. After overnight, coating buffer was removed and the wells were washed three times with washing buffer (1× PBS with 0.05% Tween-20). 200 µL 1× ELISPOT diluent was added to each well and incubated at room temperature for 1 h. The wells were washed three times with washing buffer. 100 µL of the culture medium collected above, IL-6 standards and TNF-α standards were added to each well and incubated at room temperature for 2 h. After that, samples and standards were removed, and the wells were washed three times with washing buffer. 100 µL of 1× ELISPOT diluent containing IL-6 and TNF-α detection antibody was added to each well and incubated at room temperature for 1 h. After 1 h, detection antibody solutions were removed, and the wells were washed three times with wash buffer. 100 µL of 1× ELISPOT diluent containing streptavidin-HRP was then added to each well and incubated at room temperature for 30 min. Streptavidin-HRP solution was removed, and the wells were washed five times with wash buffer. 100 µL of 1× TMB solution was added to each well and incubated at room temperature for 15 min. The reaction was stopped with by adding 50 µL of 1 M phosphoric acid to each well, and absorbance was measured at 450 nm with a SpectraMax M2e microplate reader. The concentrations of IL-6 and TNF-α in the culture medium were quantified using standard curves.

### Statistical analysis

To assess the effect of CD200 coating on macrophage phagocytosis and inflammatory cytokine secretion, unpaired t-test was used to compare unmodified particles and CD200-coated particles treated groups. Data were presented as mean ± standard deviation from three independent experiments. *p* < 0.05 was considered statistically significant.

## Results and Discussion

### Expression and purification of CD200-SA fusion protein

The gene sequences encoding CD200ECD and coreSA from pMZ006 cloned by PCR were verified by DNA gel electrophoresis with ~0.6 kb for CD200ECD and ~0.5 kb for coreSA (shown in Fig. 1b). Given the sequence of CD200ECD (amino acids 31–232, Gln-Gly, being 600 bp (~22.2 kDa), the gap (from EcoRI to BamHI) being 6 bp (~0.22 kDa), and coreSA sequence being 424 bp (~15.5 kDa, Glu-Ser), CD200-SA fusion protein has an estimated molecular weight around 38 kDa. The gene sequences of hCD200 and coreSA from PMZ006 were amplified by PCR, and the PCR products were characterized by DNA gel electrophoresis (Fig. 1b). The CD200ECD PCR product showed the size of ~0.6 kb, and coreSA PCR product showed the size of ~0.5 kb on agarose gel, confirming both gene sequences were present in pMZ006.

**Figure 2:**
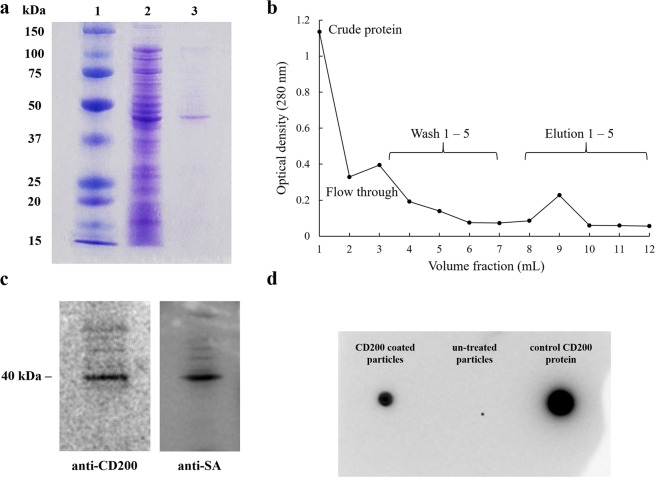
(**a**) Purification of human CD200-SA protein using HisPur Ni-NTA resin. (1) All blue protein standard; (2) crude protein lysate of human CD200-SA using Rosetta competent *E. coli* cells, induced by 0.4 mM IPTG at 37 °C for 2 h; (3) purified human CD200-SA protein. (**b**) Elution profile of CD200-SA fusion protein purified by Ni-NTA resin. Wash buffer contains 10 mM imidazole and elution buffer contains 250 mM imidazole. (**c**) Western blot of purified protein human CD200-SA with human/mouse CD200 antibody (left) and anti-streptavidin antibody (right) as primary antibody. (**d**) Dot blot analysis of CD200 coated 0.56 µm particles (left), unmodified 0.56 µm particles (middle) and control CD200 protein (right) using human/mouse CD200 antibody as primary antibody. The original SDS-PAGE gel image and Western blot image are provided in the Supplementary inforamtion file.

As shown in Fig. 2a, SDS-PAGE analysis of cell lysates indicated that the enhanced protein bands with MW of 38–40 kDa were observed for transformed bacteria with IPTG induction. The purified CD200-SA protein from IMAC columns was also characterized by SDS-PAGE along with the crude protein lysate induced at 0.4 mM IPTG for 2 h at 37 °C. The eluted protein band at ~40 kDa (shown in lane 3 of Fig. 2a) was close to the estimated size of CD200-SA fusion protein. The target protein was eluted with 250 mM imidazole elution buffer, and the elution profile showed that the target CD200-SA protein was eluted mostly in the second fraction of the elution (i.e., fraction 9 in Fig. 2b). The concentration of this eluted fraction was detected to be 187.2 ± 16.8 μg mL^−1^ (mean ± standard deviation, n = 3) by the BCA assay. The purified CD200-SA protein was further examined by Western blot analysis. As shown in Fig. 2c, the purified fusion protein was detected by both human/mouse CD200 antibody and anti-streptavidin antibody. Furthermore, CD200-coated particles (size = 0.56 µm) were characterized by dot blot analysis, with unmodified particles as negative control and CD200 protein as positive control (Fig. 2d). Dot blot analysis using human/mouse CD200 antibody confirmed the attachment of CD200 protein on biotin coated particles. The dot for CD200-coated particles was a little smaller than that for control CD200 protein probably because the diffusion of nanoparticles on PVDF membrane was not as fast as the soluble protein.

### Characterization of CD200-coated fluorescent particles

The size of biotin- and CD200-coated 0.15, 0.56, 0.84 and 2 µm polystyrene particles determined by dynamic light scattering (DLS) was shown in Table 1. The diameter of polystyrene particles increased about 18–61% after CD200 coating. Since the size increments of CD200-tagged particles are not significant, there is no aggregation of biotinylated particles via four biotin-binding sites on CD200-streptavidin. This is most likely due to the orientation and steric hindrance of CD200-SA limiting the biotin-SA binding to single particle. As shown in Table 1, the polydispersity index (PDI) of 0.15, 0.56, 0.84 and 2 µm polystyrene particles ranged from 0.207 to 0.327. After CD200 coating, the PDI increased slightly spanning from 0.223 to 0.345. Since the particle size distributions were all pretty uniform, it is assured that there was no notable aggregation of CD200-coated particles. As regards to the stability test, all 4 different sizes of CD200-coated particles after 72-h incubation at 4 °C had the sizes and PDIs similar to the ones listed in Table 1, indicating CD200-coated particles were relatively stable within 3 days of incubation. The zeta potential of all four unmodified particles was negative, ranging from −57 to −30 mV. After CD200 coating, all of the four polystyrene particles became less negatively charged, with zeta potential ranging from −18 to −9 mV. This is probably because CD200-SA protein is less negatively charged than biotinylated polystyrene particles. With less negatively charged surface of CD200-coated particles and sufficient amount of CD200 molecules coated, we believe the underlying particle chemistry (e.g., surface charge) has a minimal effect compared to CD200 exerted on macrophagic uptake of particles.

**Table 1:** Size, polydispersity index (PDI) and zeta potential of unmodified and CD200-coated particles.

Size of particles provided by vendor	Unmodified polystyrene particles			CD200-coated polystyrene particles		
	*Size (nm)*	*Polydispersity index (PDI)*	*Zeta potential (mV)*	*Size (nm)*	*Polydispersity index (PDI)*	*Zeta potential (mV)*
0.15 µm	289.29 ± 4.73	0.207 ± 0.015	−30.1 ± 2.88	347.35 ± 10.61	0.223 ± 0.071	−13.25 ± 3.87
0.56 µm	983.16 ± 52.17	0.257 ± 0.142	−41.30 ± 1.87	1583.32 ± 131.77	0.280 ± 0.015	−9.94 ± 2.85
0.84 µm	1573.22 ± 103.42	0.306 ± 0.005	−57.85 ± 5.13	2152.24 ± 268.96	0.312 ± 0.099	−18.43 ± 1.90
2.00 µm	3527.93 ± 254.26	0.327 ± 0.083	−42.07 ± 2.20	4162.70 ± 570.72	0.345 ± 0.073	−15.83 ± 0.82

### The effect of CD200 on phagocytosis of THP-1 macrophages

**Figure 3:**
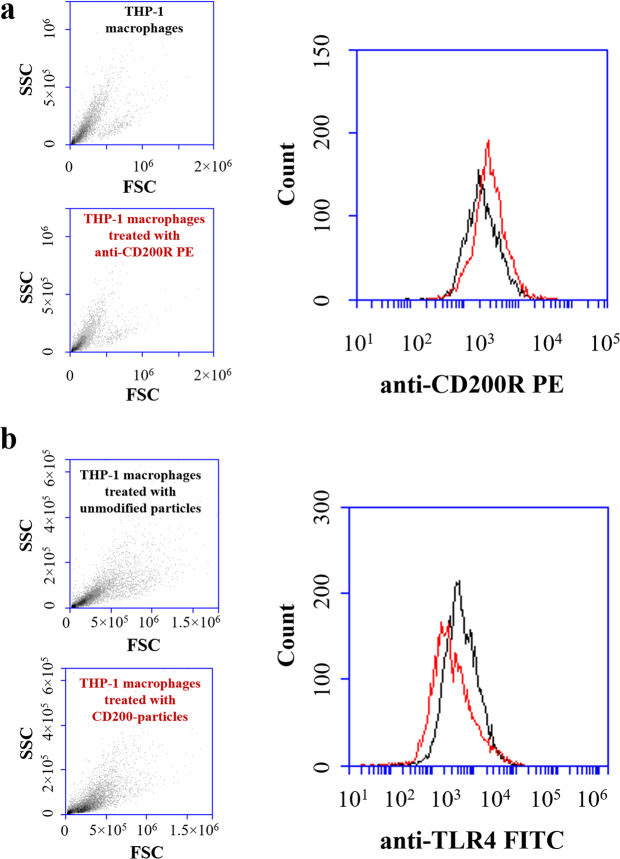
(**a**) The expression level of CD200R on THP-1 macrophages was analyzed by FACS. THP-1 macrophages were treated with anti-human CD200R with PE label. Non-treated macrophages were used as control. The MFI of untreated cells and anti CD200R-PE treated cells were 1,031 ± 83 and 1,266 ± 109, respectively. Data were presented as mean ± standard deviation (n = 3). (**b**) FACS analysis of THP-1 macrophages engulfing control and CD200 coated 0.56 µm polystyrene particles for 2 h. FACS analysis showed that CD200 coated particles decreased TLR4 expression on THP-1 macrophages. The MFI of untreated cells and anti TLR4-FITC treated cells were 8,409 ± 917 and 3,965 ± 324, respectively. Data were presented as mean ± standard deviation (n = 3).

**Figure 4:**
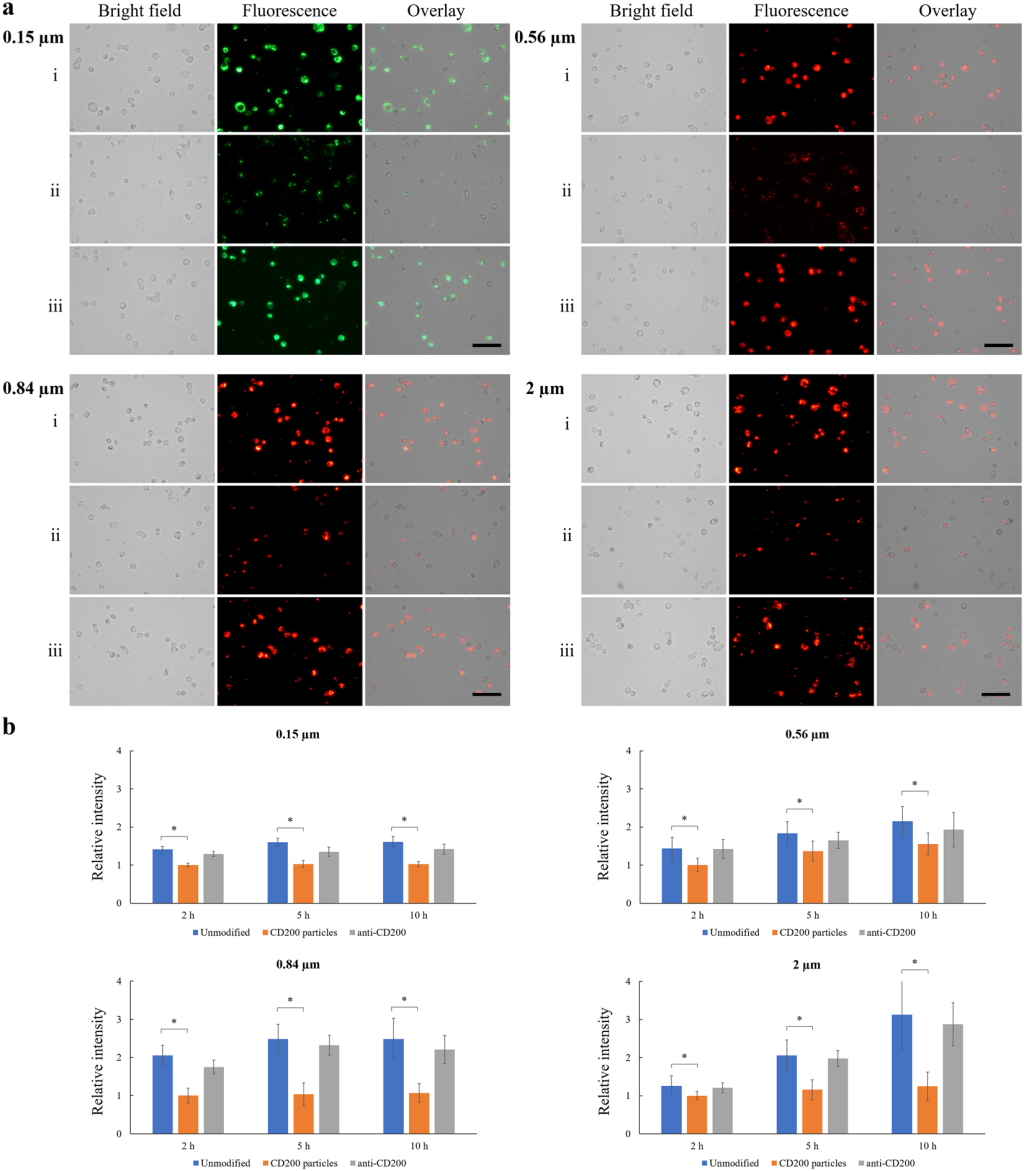
(**a**) Bright field, fluorescence and overlay images of THP-1 macrophages engulfing (i) unmodified fluorescent polystyrene particles, (ii) CD200-coated fluorescent polystyrene particles, and (iii) CD200-coated fluorescent polystyrene particles blocked with anti-CD200 antibody. Cells were incubated with 0.15, 0.56, 0.84 µm and 2 µm polystyrene particles for 5 h. Scale bar denotes 100 μm. (**b**) Relative fluorescence intensity of THP-1 macrophages engulfing unmodified fluorescent particles, CD200-coated fluorescent particles, and CD200-coated fluorescent particles blocked with anti-CD200 antibody at 2, 5 and 10 h post-treatment. Data were presented as mean ± standard deviation (n = 3). *Denotes *p* < 0.05.

**Figure 5:**
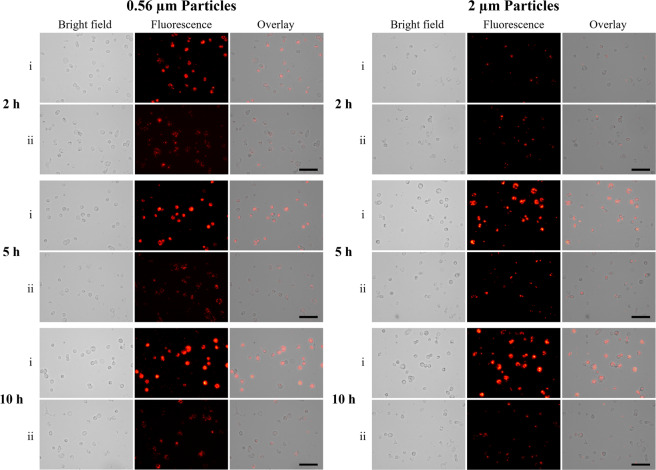
Bright field, fluorescence and overlay images of THP-1 macrophages engulfing (i) unmodified and (ii) CD200-coated fluorescent polystyrene particles (0.56 and 2 µm) at 2, 5 and 10 h post-treatment. Scale bar denotes 100 μm.

First, the level of CD200R expression on THP-1 macrophages was analyzed by FACS. The mean fluorescence intensity (MFI) slightly increased from 1,031 to 1,266 compared to the background control (i.e., no binding with anti-CD200R-PE) after THP-1 macrophages were treated with anti-CD200R-PE (Fig. 3a). Our results indicated that the expression level of CD200R on THP-1 macrophages is low, which is similar to the findings of Byrareddy *et al*.^25^. To assess the effect of CD200 on macrophage phagocytosis, THP-1 macrophages were loaded with unmodified and treated fluorescent polystyrene particles, ranging from 0.15 to 2 µm for 5 h (Fig. 4a). The fluorescent intensities of unmodified particles (group i) and CD200-coated particles blocked with anti-CD200 antibody (group iii) for all four different sizes ingested by macrophages were significantly higher than the ones of CD200-coated counterparts (group ii). From the bar charts presented in Fig. 4b, the unmodified particles (group i) had already been taken up by the THP-1 macrophages at 2 h. The fluorescence intensity did not increase much after 5 and 10 h for 0.15, 0.56 and 0.84 μm particles, indicating most of the nano-sized particles had been taken up by the macrophages within 2 h. For CD200-coated particles with all four sizes (group ii), CD200 acting through CD200R did suppress the phagocytic process of macrophages. The phagocytosis-resistant efficacy of CD200 retained up to 10 h. The fluorescence intensity of group iii was slightly lower than group i, and significantly stronger than group ii (Fig. 4b), supporting the notion that reduced phagocytic activity was due to CD200-CD200R interaction. For comparison, cellular images of THP-1 macrophages engulfing nanoparticles (i.e., 0.56 µm) and microparticles (i.e., 2 µm) at 2, 5, and 10 h were shown in Fig. 5. It was noticed that 2 µm microparticles were taken up much slower than the other three types of particles. They were not engulfed by the cells until 5–10 h (Fig. 5), probably because the large size of microparticles delayed the phagocytosis process.

**Figure 6:**
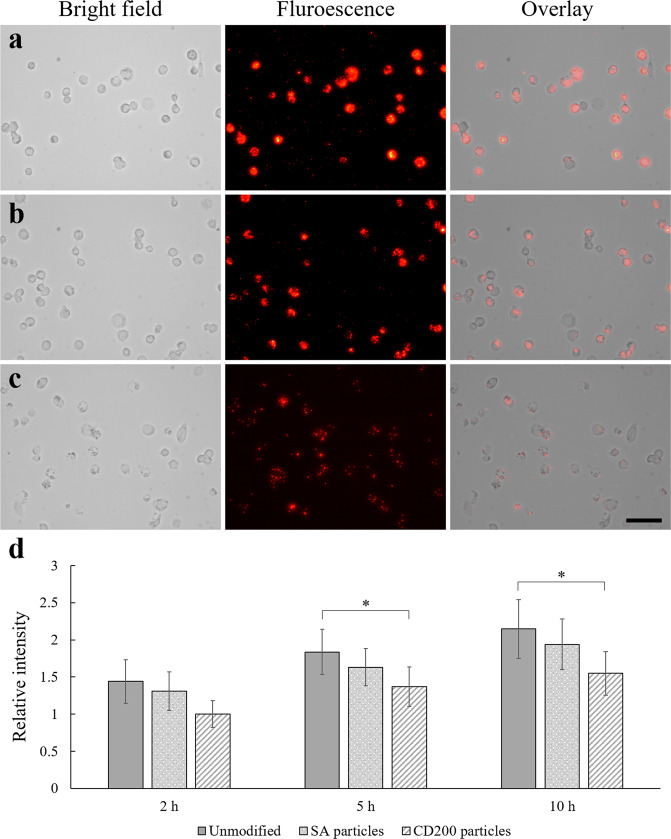
THP-1 macrophages were allowed to engulf (**a**) unmodified (**b**) SA coated (**c**) CD200-SA coated 0.56 µm polystyrene particles for 5 h. Scale bar denotes 100 µm. (**d**) Relative fluorescence intensity of THP-1 macrophages engulfing unmodified, SA-coated and CD200-coated 0.56 µm polystyrene particles at 2, 5 and 10 h post-treatment. Data were presented as mean ± standard deviation (n = 3). *Denotes *p* < 0.05.

To further confirm the anti-phagocytosis effect was caused solely by CD200 (rather than partial effect from the fused SA section), THP-1 macrophages were treated with control, SA-coated, and CD200-SA coated 0.56 µm polystyrene particles. It should be noted that all four sizes of CD200-tagged particles (0.15, 0.56, 0.84 and 2 µm) tested in this study revealed the same trend of diminishing phagocytosis. Instead of showing all sizes with lots of images, 0.56 µm was chosen as the representative particles for this study to illustrate the trend also observed for the other examined particle sizes. After 5 h, most unmodified and SA-coated particles were taken up by THP-1 macrophages (Fig. 6a,b). Quantification of the fluorescence intensity showed the amount of SA-coated particles taken up by macrophages was 16.4% lower than unmodified particles. For particles coated with CD200-SA, the amount of particles taken up by macrophages was decreased by 44.7% (Fig. 6c). The effect of streptavidin-coated particles on phagocytosis after 2, 5, and 10 h incubation was also shown as a bar chart (i.e., Fig. 6d) in comparison with biotinylated and CD200-coated particles. The results indicated that the diminished phagocytosis of particles was indeed attributed to CD200 which was engaged with CD200R to inhibit the activation of macrophage phagocytosis, rather than the attribution of coreSA protein.

In a previous study, CD200-deficient mice showed accelerated macrophage activation due to the absence of CD200-CD200R interaction^13^. Increased phagocytosis was also reported for microglial cells isolated from CD200-deficient mice in another study^21^. On the contrary, administration of soluble CD200-Fc fusion protein restrained phagocytosis of oligodendrocyte precursor cells by peritoneal macrophages^20^. These studies all suggest that CD200-CD200R engagement plays an inhibitory role in macrophage phagocytosis. One possible explanation for this functional finding is that CD200 deficiency increases expression of TLR4 on microglia^21^, which can result in enhanced phagocytosis; however, soluble CD200 downregulates TLR4 expression on macrophage, leading to reduced phagocytosis. In the present study, to check whether CD200 regulates TLR4 expression on THP-1 macrophages, FACS analysis was performed on cells engulfing control particles and CD200-coated particles. As shown in Fig. 3b, a higher MFI was observed on THP-1 macrophages challenged with untreated particles (MFI = 8,409). When THP-1 macrophages were treated with CD200-coated particles, the MFI dropped to 3,965 indicating that CD200 coating decreased TLR4 expression on THP-1 macrophages. Our results were consistent with the finding by Hayakawa *et al*.; when macrophages (isolated from rat peritoneal cavity) were treated with soluble CD200-Fc, surface levels of TLR4 were downregulated^20^.

**Figure 7:**
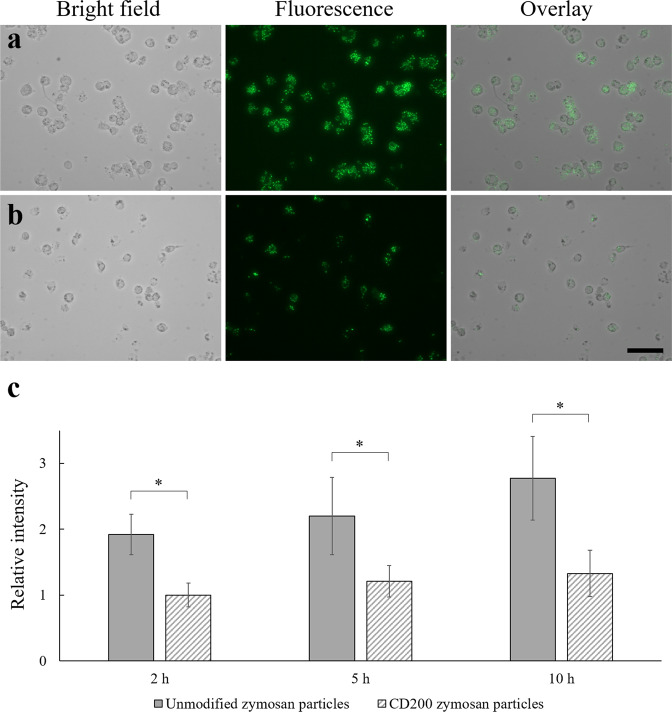
Bright field, fluorescence and overlay image of THP-1 macrophages engulfing (**a**) FITC-zymosan particles and (**b**) CD200-coated FITC-zymosan particles for 5 h. Scale bar denotes 100 μm. (**c**) Relative fluorescence intensity of THP-1 macrophages engulfing unmodified and CD200-coated FITC-zymosan particles at 2, 5 and 10 h post-treatment. Data were presented as mean ± standard deviation (n = 3). *Denotes *p* < 0.05.

We further examined if the material used to make the particles would affect the observed anti-phagocytic feature of CD200-coated latex particles. Zymosan particles comprised of yeast wall were selected for this study. Generally, macrophage cell lines express TLRs such as TLR2, TLR4, TLR6 on their surfaces^26,27^. Macrophage phagocytosis activities can be stimulated by zymosan through TLR2-mediated signaling^28^. This will upregulate the expression of TLR2 and lead to increased secretion of inflammatory cytokines such as TNF-α and interleukin-8 (IL-8)^29^. As shown in Fig. 7a,b, THP-1 macrophages challenged with CD200-coated FITC-zymosan particles (average size of 3 μm) exhibited much less phagocytosis than the ones treated with control FITC-zymosan particles up to 5 h. This result implied that the signal elicited by CD200-CD200R engagement could down-regulate TLR4 but also attenuated phagocytosis stimulated by TLR2 involved with zymosan. It should be noted that after 2-h treatment, the amount of unmodified zymosan particles (3 µm) engulfed by THP-1 macrophages was much higher than the unmodified 2 µm polystyrene particles. The fluorescence intensity readings also confirmed that 3 μm zymosan particles were taken up by THP-1 macrophages faster than the 2 µm polystyrene particles (Fig. 7c); the macrophages started to uptake zymosan particles at 2 h, while they started to uptake polystyrene particles after 5 h (Fig. 4b). By comparing these two types of particles with similar sizes, our results showed that zymosan particles stimulated the phagocytic activity of THP-1 macrophages within 2 h of incubation.

Our findings and other aforementioned studies from other research groups suggest that the presence of interaction between CD200 and CD200R diminishes the engulfment capability of phagocytes. However, Chen *et al*. coated micro-sized poly(lactic-co-glycolic acid) (PLGA) particles with CD200 and found that CD200 enhanced phagocytosis of PLGA particles (average size of 7 µm) by human monocytes and murine macrophages^22^. The PLGA microparticles were synthesized and conjugated with CD200-PE by that group. As noted in their study, high heterogeneity of CD200 conjugation was observed varying from particle to particle. With their determined average density of 30 molecules µm^−2^, the heterogeneous distribution of CD200 on 7-µm microparticles might lead to extremely low number of molecules encountering CD200R expressed on macrophages and monocytes used in the study, and thereby not triggering antiphagocytic potency from the engagement of CD200-CD200R. Macrophages generally engulf particles with size less than 12 µm^30^. However, the phagocytic activity of macrophages decreases when particle size increases^31^. PLGA particles with average size of 7 µm most likely could render less phagocytosis activities than particles of smaller sizes. Indeed, Chen *et al*. reported that only 8% of cells phagocytosed control PLGA microparticles and 15% of cells engulfed CD200-PLGA particles after 12-h incubation. Due to extremely low phagocytic rate, the claim of 2-fold increment of phagocytosis of CD200-coated PLGA microparticles could be a concern. It has been reported that microparticles with size 2–3 µm are optimal for macrophage phagocytosis^32,33^. Furthermore, nanoparticles are generally delivered easier to a greater portion of a macrophage population^34^. In this study, the sizes of control particles (including polystyrene and zymosan) ranged from 0.15 to 3 µm. Based on equation [1], the calculated percentage of cells phagocytosed untreated micro/nanoparticles was in the range of 70–90% (the fluorescence was qualitatively shown in Fig. 4a). With such high rate of phagocytosis, the antiphagocytic effect attributed to CD200-CD200R interaction could be firmly determined. Since the biotinylated fluorescent polystyrene particles in this study were purchased from a vendor with much uniform distributions of fluorescence and biotin, there was no concern of heterogeneity of CD200 in contact with CD200R on macrophages. To quantitatively determine the amounts of CD200-SA molecules coated on the biotinylated particles, we assumed that only one binding site of streptavidin bound with one biotin molecule on the polystyrene particles. After one CD200-SA molecule binds one biotin molecule anchored on the particle, there are three SA sites remaining for biotin binding. Hence, three biotin-FITC molecules can bind CD200-SA molecule. Once the fluorescence of FITC was measured to obtain the number of molecules of biotin-FITC via a pre-determined calibration curve, the number of CD200 molecules tagged on the particle is equal to one third of the number of biotin-FITC molecules. Based on the assumption, the surface density of CD200 coated on the particle was calculated to be 151 ± 23, 564 ± 86, 846 ± 163 and 2,015 ± 442 molecules µm^−2^ for 0.15, 0.56, 0.84 and 2 µm polystyrene particles, respectively. The amount of CD200 coated on particles should be sufficient over the threshold value to engage with CD200R and lead to reduced phagocytosis.

### The effect of CD200 on IL-6 and TNF-α secretion from THP-1 macrophages

**Figure 8:**
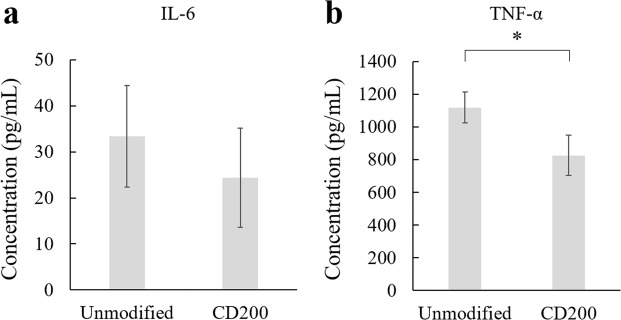
THP-1 macrophages were allowed to engulf unmodified and CD200 coated 0.56 µm polystyrene particles for 18 h. After 18 h, culture media were collected and concentrations of (**a**) IL-6 and (**b**) TNF-α were measured by commercial ELISA kits. Data were presented as mean ± standard deviation (n = 3). *Denotes *p* = 0.006.

Macrophages can generally be activated by treating cells with lipopolysaccharide (LPS) or interferon-gamma (IFN-γ). However, in this study, THP-1 macrophages were not activated to simplify the experiment. The effect of CD200 coating on level of pro-inflammatory cytokine IL-6 and TNF-α secretion was shown in Fig. 8a. Macrophages treated with CD200-coated 0.56 µm nanoparticles secreted 26.1% less TNF-α than unmodified nanoparticles (Fig. 8a). The level of TNF-α detected in the present study was much higher than IL-6 detected. Although THP-1 macrophages treated with CD200-coated nanoparticles secreted 26.9% less IL-6 than unmodified nanoparticles, the IL-6 secretion level of was low to start with (~30 pg mL^−1^) (Fig. 8b). In this study, the inhibition of IL-6 and TNF-α secretion level was not as dramatic as previously reported by Kim *et al*.^35^, probably because the THP-1 macrophages in the study were not activated. The level of pro-inflammatory cytokines, especially IL-6, was low to start with. The difference of IL-6 between control group and CD200 treated group was not statistically significant. Compared to literature, Pietila *et al*. reported 1763.5 pg mL^−1^ TNF-α secreted from unactivated THP-1 macrophages, and it increased to 5002.3 and 2510.3 pg mL^−1^ after activation by LPS and IFN-γ, respectively^36^. Li *et al*. reported low production of IL-6 from THP-1 macrophages (~50 pg mL^−1^)^37^, which was similar to the findings of this study. In short, our findings indicated that coating of CD200 on nanoparticles decreased the level of TNF-α secretion from THP-1 macrophages.

## Conclusions

A plasmid encoding CD200 extracellular domain and coreSA was constructed. CD200-SA fusion protein was expressed by bacterial transformation and IPTG induction. Surface modification of CD200 reduced macrophage phagocytosis of polystyrene particles ranging from 0.15 to 2 µm and zymosan particles with size of 3 µm. The expression of TLR-4 on THP-1 macrophages was down-regulated after treating cells with CD200-coated particles. Secretion of TNF-α from THP-1 macrophages was decreased when cells were treated with CD200-coated particles compared to unmodified particles. Finally, it should be noted that our findings are based merely on THP-1 induced macrophages. Further study is needed to examine if antiphagocytic and immunoregulatory effects of immobilized CD200 reported here also apply with primary macrophage cells.

## Supplementary information

Supplementary information.
