# Supplementary material for: Diminution of Phagocytosed Micro/Nanoparticles by Tethering with Immunoregulatory CD200 Protein
Source: Sci Rep. 2020 May 25;10:8604. doi: 10.1038/s41598-020-65559-z (PMC7248097; doi:10.1038/s41598-020-65559-z)
Supplement: Supplementary file 1 — Supplementary information. [file 41598_2020_65559_MOESM1_ESM.docx]

**Diminution of Phagocytosed Micro/Nanoparticles by Tethering with Immunoregulatory CD200 Protein**

Jun Zhang and Ching-An Peng*

Department of Biological Engineering, University of Idaho, Moscow, ID 83844, USA

*Corresponding address:

Ching-An Peng

Department of Biological Engineering

Engineering Physics Building 421

875 Perimeter Drive MS 0904

Moscow, ID 83844-0904

Phone: 208-885-7461

E-mail: [capeng@uidaho.edu](mailto:capeng@uidaho.edu)


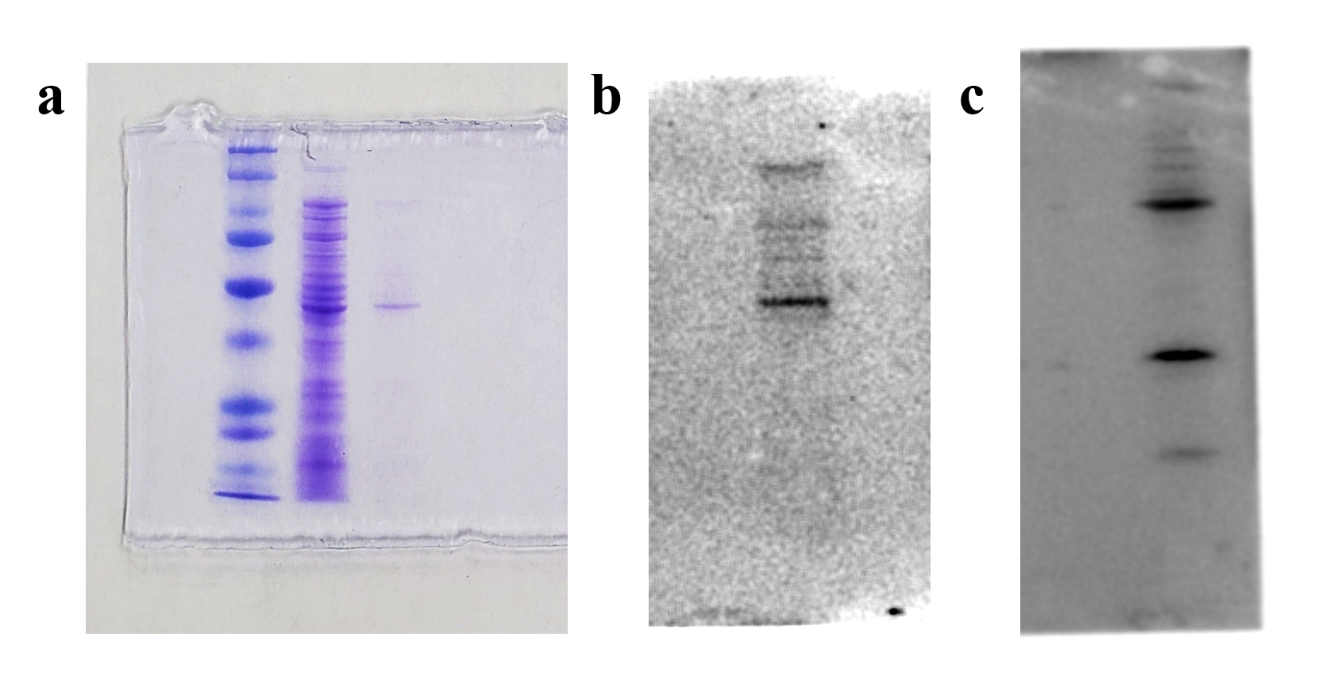


**Figure S1**. (a) SDS-PAGE gel image of protein standard (size marker), crude protein lysate of CD200-SA and purified human CD200-SA protein was performed in one gel. (b) Western blot image of purified CD200-SA protein using human/mouse CD200 antibody as primary antibody and mouse IgG HRP-conjugated antibody as secondary antibody. (c) Western blot image of purified CD200-SA protein using anti-SA antibody as primary antibody and goat anti-rabbit IgG horseradish peroxidase conjugate as secondary antibody. Western blots in (b) and (c) were performed on different blots because different primary and secondary antibodies were used.
